# Overactive EGF signaling suppresses a* C. elegans* *pnc-1* egg-laying phenotype independent of known signaling mediators.

**DOI:** 10.17912/micropub.biology.000482

**Published:** 2021-10-04

**Authors:** Matt Crook, Wendy Hanna-Rose

**Affiliations:** 1 Texas A&M University-San Antonio; 2 The Pennsylvania State University

## Abstract

Nicotinamide recycling is critical to the development and function of *Caenorhabditis elegans*. Excess nicotinamide in a *pnc-1* nicotinamidase mutant causes the necrosis of uv1 and OLQ cells and a highly penetrant egg laying defect. An EGF receptor (*let-23*) gain-of-function mutation suppresses the Egl phenotype in *pnc-1* animals. However, gain-of-function mutations in either of the known downstream mediators, *let-60/ Ras* or *itr-1*, are not sufficient. Phosphatidylcholine synthesis is neither required nor sufficient, in contrast to its role in the *let-23gf* rescue of uv1 necrosis. The mechanism behind the *let-23gf* suppression of the *pnc-1 *Egl phenotype is unknown.

**Figure 1:**
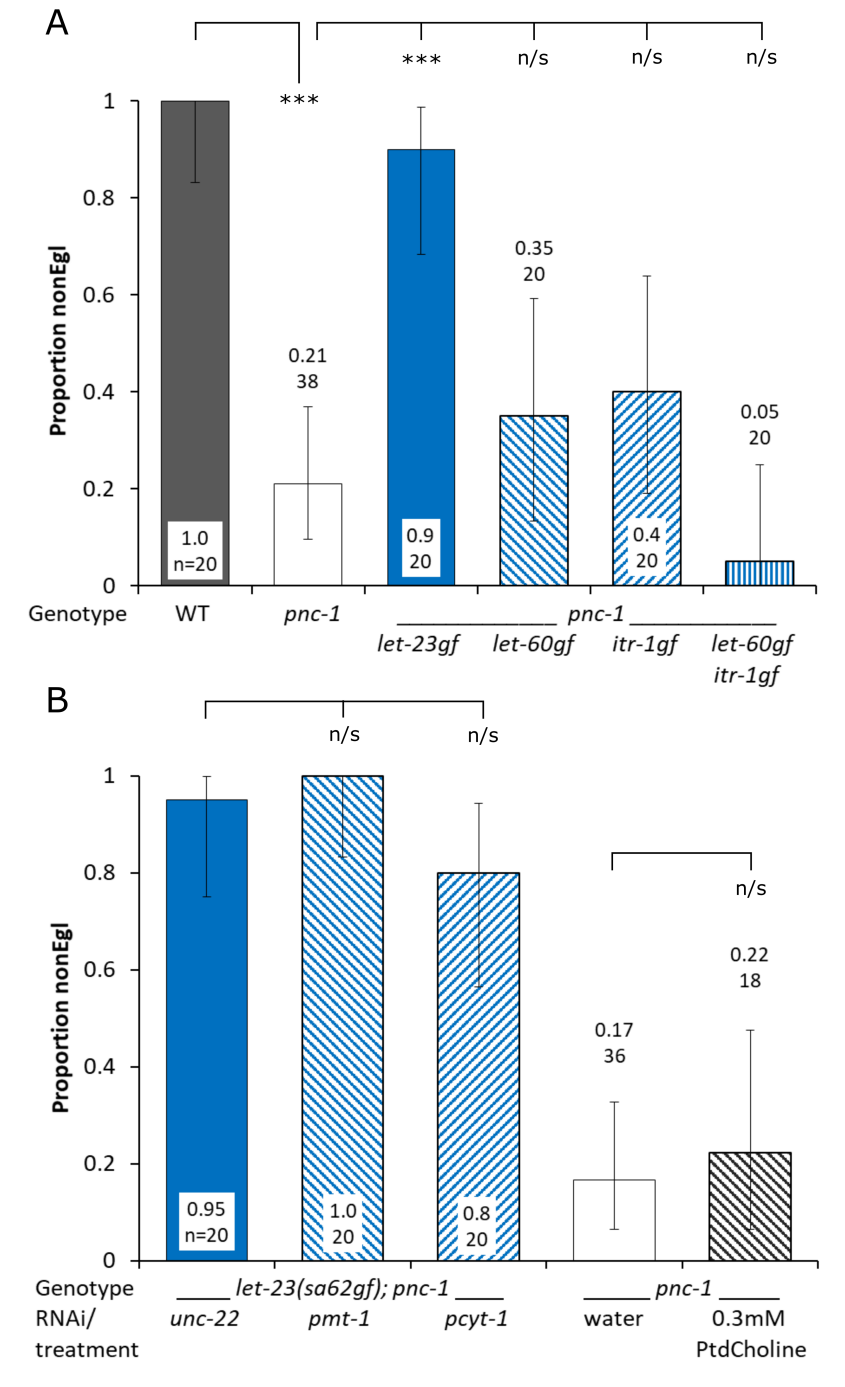
A *let-23(sa62)gf* mutation in *C. elegans* strongly suppresses a *pnc-1(pk9605)* egg-laying (Egl) phenotype, where adult *pnc-1(pk9605)* hermaphrodites form “bags of worms” by four days post L4>adult molt. A) A gain-of-function mutation in *let-23* is sufficient to rescue the *pnc-1* Egl phenotype, but gain-of-function mutations in downstream genes *let-60* and/ or *itr-1* are not. B) Phosphatidylcholine synthesis is neither required nor sufficient for the *let-23(ga62)gf* rescue of the *pnc-1* Egl phenotype. WT = wild-type *C. elegans* N2 Bristol strain. Error bars are 95% confidence intervals, with proportion and sample size in the data labels. Proportions were analysed by pairwise.prop.test in R with Holm p value adjustment; *** and n/s represent p<0.0001 and non-significant, respectively.

## Description

NAD^+^ is an electron carrier and a co-substrate for NAD^+^-dependent enzymes such as poly(ADP-ribose) polymerases (Bouchard *et al.* 2003; Sauve 2008). The byproduct of these enzymatic reactions, nicotinamide (NAM), must be salvaged to maintain a readily available NAD^+^ pool. In *Caenorhabditis elegans* the nicotinamidase PNC-1 acts both cell autonomously and non-cell autonomously to convert NAM into nicotinic acid (NA), an NAD^+^ precursor in this organism (Huang and Hanna-Rose 2006; Vrablik *et al.* 2009; Crook *et al.* 2014). Loss of PNC-1 function affects NAD^+^ pathway metabolites in two ways. It results in an increase in NAM, causing necrosis of OLQ and uv1 cells, and an egg-laying phenotype due to reduced muscle function. It also reduces NAD^+^ levels, resulting in gonad developmental delay and a male mating defect (Huang and Hanna-Rose 2006; Vrablik *et al.* 2009; Vrablik *et al.* 2011; Upadhyay *et al.* 2016).

LET-23 is the sole *C. elegans* Epidermal Growth Factor (EGF) receptor and is involved in a range of biological and developmental processes, including vulval development and specification of the uv1 cells (Chang *et al.* 1999; Moghal and Sternberg 2003). A gain-of-function mutation in the extracellular domain, *let-23(sa62)gf*, results in precocious activation of LET-23 independent of its EGF ligand LIN-3 (Katz *et al.* 1996).Overactivation of LET-23 rescues the uv1 cell necrosis phenotype of *pnc-1* loss-of-function mutants, and this rescue requires phosphatidylcholine synthesis (Huang and Hanna-Rose 2006; Crook *et al.* 2016; Crook and Hanna-Rose 2020). We noted that the egg-laying phenotype of *pnc-1* was also ameliorated by overactivation of LET-23 and decided to investigate the mechanism.

To study the role of EGF signaling in the prevention of the egg-laying phenotype we placed individual L4 hermaphrodites on Nematode Growth Medium (NGM) agar plates spotted with *Escherichia coli* OP50. Individual animals were observed after two, three and four days at 20C and scored as non-Egg laying defective (nonEgl) adults or “bags of worms” (Egl), where larvae hatch in the uterus due to a failure to lay eggs. Proportion nonEgl was calculated as the number nonEgl adults/ total number of individuals at day four. All nonEgl adults had laid eggs by day 4. We found that the *pnc-1(pk9605)* loss-of-function allele reduced the proportion of nonEgl adults to 0.21 and that the *let-23(sa62)* gain-of-function (gf) allele in a *pnc-1(pk9605)* background restored that to 0.9 (Fig. 1a). However, gain-of-function mutations in *let-60* or *itr-1*, which mediate signal transduction downstream of *let-23* (Clandinin *et al.* 1998; Chang *et al.* 1999), had no effect on the *pnc-1* egg-laying phenotype (Fig. 1a).

Phosphatidylcholine synthesis is required for *let-23(sa62)gf* mediated rescue of uv1 necrosis and exogenous phosphatidylcholine alone is partially sufficient for uv1 survival (Crook *et al.* 2016). PMT-1 is part of the Sequential Methylation Pathway (SMP) that synthesizes phosphocholine (Brendza *et al.* 2007), and PCYT-1 turns phosphocholine from the Sequential Methylation and Kennedy pathways into CDP-choline, the precursor of phosphatidylcholine (Kennedy and Weiss 1956). To test if phosphatidylcholine synthesis was required for the *let-23(sa62)gf*-mediated rescue of the *pnc-1* egg-laying phenotype we knocked down *pmt-1* or *pcyt-1* by RNAi. *unc-22* (control), *pmt-1* and *pcyt-1* RNAi bacterial cultures were spotted onto NGM plates containing 50 μg.ml^-1^ ampicillin and 1 mM IPTG, then individual L4 hermaphrodites were added to each plate and scored as above. We found that neither *pmt-1* nor *pcyt-1* were required for rescue in a *let-23(sa62)gf; pnc-1(pk9605)* background (Fig. 1b). *pmt-1* or *pcyt-1* RNAi did however reduce uv1 cell survival in nonEgl adults in experiments run concurrently with this project (Crook *et al.* 2016), suggesting that RNAi knockdown of the target genes was effective. Next, we wanted to see if phosphatidylcholine alone was sufficient for rescue, as it ameliorates the uv1 necrosis phenotype (Crook *et al.* 2016). We supplemented *pnc-1* animals with 0.3 mM phosphatidylcholine but found no effect on the *pnc-1* egg-laying phenotype (Fig. 1b).

We have shown that overactivation of the *C. elegans* *let-23* EGF receptor robustly rescues the *pnc-1* egg-laying phenotype, but that gain-of-function mutations in the known downstream signaling mediators *let-60/ Ras* and *itr-1* are not sufficient. Phosphatidylcholine synthesis is not required for the *let-23(sa62)gf* rescue of the egg-laying phenotype and phosphatidylcholine supplementation of *pnc-1* had no significant effect at the sample sizes used, in contrast to the role of phosphatidylcholine in *let-23(sa62)gf* rescue of uv1 necrosis. We have clearly demonstrated another role for *let-23* outside that of growth and development. However, the mechanism by which overactive LET-23 rescues egg-laying in *pnc-1* animals is not clear. LET-23 may act *via* an as yet unknown pathway that restores uterine or vulval muscle function by either reducing the production of nicotinamide in those tissues or promoting some other compensatory mechanism.

## Reagents

Strains:

N2 Bristol

BL5715 *inIs179 (ida-1::gfp)* II

HV560 *inIs179 (ida-1::gfp)* II*; pnc-1(pk9605)* IV

HV639 *inIs179 (ida-1::gfp)* II*; pnc-1(pk9605) let-60(n1046gf) itr-1(sy290gf) unc-24(e138)* IV

HV662 *inIs179 (ida-1::gfp)* II*; pnc-1(pk9605) let-60(n1046gf)* IV

HV663 *inIs179 (ida-1::gfp)* II*;* *pnc-1(pk9605) itr-1(sy290gf)* *unc-24(e138)* IV

HV776 *let-23(sa62gf) inIs179(ida-1p::gfp)* II*; pnc-1(pk9605)* IV

The strains used in this study are available from the authors upon request.

We used the following clones from the Ahringer RNAi library: *pmt-1* ZK622.3 II-4G04, *pcyt-1* F08C6.2 X-3N20, *unc-22* ZK617.1 IV-6K06.
